# Content and Duration of Doctor-Patient Communication in Outpatient Oncology Follow-Up Consultations in China

**DOI:** 10.7759/cureus.55597

**Published:** 2024-03-05

**Authors:** Yinxia Zhan, Peiyang Mao, Feng Gao, Qiuling Shi

**Affiliations:** 1 School of Public Health, Chongqing Medical University, Chongqing, CHN; 2 Department of Oncology, Mianyang Central Hospital, School of Medicine, University of Electronic Science and Technology of China, Mianyang, CHN

**Keywords:** cancer patients, china, consultation duration, outpatient follow-up, oncology

## Abstract

Purpose

This study aimed to analyze the content of counseling between cancer patients and physicians during outpatient follow-up, assess the duration of different communication content, and explore the influencing factors.

Patients and methods

We observed consecutive cancer patients without a first diagnosis of malignancy who presented to the oncology outpatient clinic of a public hospital from October through December 2023. A structured observation form was used to record the content and duration of doctor-patient communication. All variables were electronically recorded and stored on a data management platform. Multiple linear regression was used to examine the determinants of consultation duration.

Results

Our study included 620 cancer patients, of whom 281 individuals had distant metastasis. The average consultation duration for 620 cancer patients was 4.85 minutes (standard deviation of 3.09 minutes). The category of doctor-patient communication was routine outpatient follow-up, involving 182 patients, with an average consultation duration of 3.81 minutes (standard deviation of 2.24 minutes). The main communication content for 51 patients was about the admission procedures, with an average consultation duration of 3.00 minutes (standard deviation of 1.92 minutes). For 67 patients, the primary communication involved the prescription of anti-tumor medications, with an average consultation duration of 3.70 minutes (standard deviation of 2.30 minutes). Symptom control discussions were the main communication content with 107 patients, with an average consultation duration of 5.91 minutes (standard deviation of 3.44 minutes). The main communication content of 24 patients involved medical insurance reimbursement, with an average duration of 7.75 minutes (standard deviation of 4.63 minutes). Consultations accompanied by caregivers and the presence of metastatic tumors were positively associated with consultation duration, whereas female patients with genital malignancies were negatively associated with consultation duration. Communication about symptom management and communication about medical insurance reimbursement was positively associated with the duration of medical consultations. Communication about administering the formalities requisite for patient admission was negatively associated with the consultation duration.

Conclusion

We classified the main contents of doctor-patient communication in the oncology outpatient clinic and found that the contents of doctor-patient communication tended to be monotone and systematized. Oncology outpatient models should consider the integration of caregiver involvement. The reason for the shorter outpatient consultation duration in female patients of cancer primary in the reproductive system needs further investigation. The intricate medical insurance reimbursement process poses an increasing time challenge for outpatient clinicians. In addition, the adequacy and effectiveness of symptom management services provided to cancer patients during oncology outpatient follow-up require further evaluation and review.

## Introduction

As the number of cancer patients increased, the focus of follow-up shifted to the management of chronic disease [1]. They are tightly linked to hospitals to obtain continued support and information [2]. Outpatient visits help identify physical and emotional complications that may emerge months or years post-treatment completion. The physiological and psychological symptoms associated with treatment present an enduring challenge to cancer patients.

Cancer treatments are expensive, and patients possess varying degrees of economic burden [3]. In the United States, the front desk staff verifies patients' insurance eligibility and coverage before consultation commencement. The practice management system in China is notably different from that of the European Union and the United States. The verification and the claims process are regularly performed by physicians during patient consultations [4]. This limits the patients' time to report their experiences related to disease and treatment.

These situations highlight the challenges of giving patients the necessary time to express their agenda during these limited consultations [5]. Studies have established that longer consultation lengths are associated with enhanced physician-patient communication, improved medical service quality, better quality of life, and more favorable health outcomes [6]. In many cases, it is impractical for oncologists to complete comprehensive consultations during these short consultations, let alone individualized medical services for patients [7]. Doctor-patient communication is not just a channel for doctors to provide medical information; it is also a window for doctors to understand patients' comprehensive needs [8]. The needs of cancer patients vary individually. Analyzing the content of doctor-patient communication is not only beneficial for creating personalized care strategies but also aids in optimizing resource allocation in medical institutions and enhancing service efficiency [9]. Due to these restricted consultations, patients' needs are often not adequately met, including the management of pain and emotional needs. Various studies have indicated the possible risks associated with not identifying these patients' needs [10]. Health systems have begun to focus on improving the efficiency and appropriateness of outpatient appointments in response to the increasing needs of cancer patients.

In this study, we recorded consultation durations and various aspects of communication between cancer patients and outpatient oncologists to reflect the quality of medical care and show the real-world follow-up of oncology outpatient clinics in public hospitals. We also analyzed the factors associated with consultation durations to identify potential cancer groups with shorter consultation durations. This study is the first investigation of oncology outpatient clinics in China. It is hoped that this study can provide a reference for the outpatient management of hospitals in China in the future.

## Materials and methods

Study design

A cross-sectional study was conducted from October to December 2023 at the Oncological Outpatient Department of Mianyang Central Hospital in China. The hospital is a regional medical center in a prefecture-level city in Sichuan Province, with an annual outpatient volume of 1.27 million. At the hospital's outpatient oncology clinic, each oncologist conducts an average of one clinic per week, with each session lasting half a day. The study protocol was approved by the Biomedical Ethics Committee of Mianyang Centre Hospital (Ethical Review Grant Number: S20220357-02). Each patient was given full information about the study and was asked to provide informed consent after consultation. The investigators removed all traces of data from patients who declined to participate. This article was previously posted to the medRxiv preprint server on August 2023.

Participants

Patients were scheduled for online appointments using their smartphones. The maximum number of online bookings for the oncology outpatient clinic was restricted to 40 times during the morning (8:00 a.m. to 12:00 p.m.) and 30 times during the afternoon (2:00 p.m. to 6:00 p.m.). Patients with pathologically confirmed malignant neoplasms who utilized a smartphone for appointment scheduling understood the nature of this study and provided their consent for participation were included in this study. Individuals with incomplete registration or appointment data, those who were diagnosed with a malignant tumor for the first time at the oncology outpatient clinic during the study period and those with multiple visits during the study period were excluded from this study.

Contents of doctor-patient communication

The main content of doctor-patient communication was classified into six categories: routine outpatient follow-up evaluation, administering the formalities requisite for patient admission, prescribing anti-tumor drugs, symptom control, medical insurance reimbursement, and others. Among these, the three different communication contents of routine outpatient follow-up evaluation, administering the formalities requisite for patient admission, and prescribing anti-tumor drugs were divided into three different single categories. The symptom control content was defined as the sum of any singular category and the symptom control content. The medical insurance reimbursement content was defined as the sum of any singular category and the medical insurance reimbursement content. The other category was defined as other communication content excluding symptom control and insurance reimbursement communication content, and was usually a combination of three single categories.

Variables

We used the hospital's outpatient medical record system to collect data on patient demographics and medical information. These included the patients' age (categorized as either <60 or ≥60 years), sex, ethnicity (Han or ethnic minority), marital status (married, single, widowed, or divorced), the highest level of education attained (below senior school or high school and above), their registered residence (urban or rural), type of health insurance (Urban Resident-based Basic Medical Insurance (URBMI), Urban Employee Basic Medical Insurance, New Rural Cooperative Medical Scheme, self-financed medical care, or social insurance), presence of caregivers during consultations (yes or no), and the timing of their outpatient appointments (either in the morning, from 8:00 a.m. to 12:00 p.m. or in the afternoon, from 2:00 p.m. to 6:00 p.m.). Additionally, we collected disease-specific information, such as the primary malignant tumor region, count of chronic conditions (0, 1, 2, or ≥3), the status of distant metastasis of malignant tumors, and the type of ongoing treatment received (targeted therapies, chemotherapy, immunotherapy, radiotherapy, endocrine therapy, other treatment, or no treatment). The categorization of patients' registered residence locations was based on the area codes for urban and rural statistics provided by the National Bureau of Statistics of China, version 2023 [11]. Detailed addresses were collected and used to confirm the urban and rural codes based on their locations. We used the neoplasms code in ICD-10 to classify malignant tumor types and chronic diseases [12]. The number of chronic diseases was determined based on the diagnoses recorded in the outpatient medical record system within a one-year look-back period. The treatment types were classified based on the Chinese Society of Clinical Oncology Guidelines for the Diagnosis and Treatment of Common Malignant Tumors, version 2023 [13]. Variables including the physician's sex and years of outpatient experience (<10 years, 10-20 years, or >20 years) were also included in the analysis.

Outcome

The primary outcome was the consultation duration. The initiation of the consultation was defined as the moment at which the patient commenced interaction with the physician, whereas its conclusion was marked by the moment at which the interaction between the patient and the outpatient physician ended. The duration of the consultation was defined as the temporal difference between these two events. The commencement and conclusion times of the consultations were meticulously recorded on a data management platform, employing a date format encompassing the year, month, day, hour, and minute. Instances with a duration of <1 minute were standardized and accounted for one full minute.

Data collection procedure

The data collection was conducted at the offices of eight outpatient physicians (Appendix 1). The data collectors logged into the hospital's outpatient medical record system before the start of the outpatient hours (8 a.m. or 2 p.m.) to check the medical record number and diagnosis at the oncology outpatient clinic for screening. A graduate student who had been trained before the survey provided two consecutive weeks of outpatient services to the participating oncologist. The graduate student sat in the least disturbed corner of the room and did not talk to the oncologist or the patient. We used a self-designed electronic observation form to observe the consultation directly in the consultation room and recorded the consultation content, consultation duration, and other information.

Data quality control

To minimize observation bias, firstly, the collection of data regarding the variable of consultation duration in the oncology outpatient clinic was carried out solely by a graduate student trained in survey methodology. The dates were encoded in the electronic collection form in year-month-day format. Secondly, we developed a standardized outpatient observation survey questionnaire, which was reviewed and approved by a professor from our team, and then coded into the data collection platform. During the observation process, a graduate student recorded the medical communication content in an electronic spreadsheet, which was reviewed and validated by the physician after the patient's outpatient consultation concluded. To mitigate the potential risk of inadvertently including the same individual more than once, the data management platform was populated with the hospital medical record numbers of the patients. In cases where a duplicate patient entry was identified, the platform automatically prevented subsequent data entry. Two researchers independently conducted a rigorous verification of the data's accuracy. In instances where data were missing, diligent efforts were conducted to contact patients by phone to obtain the necessary information. Missing data for this patient were removed when the patient could not be reached by telephone. Any discrepancies were meticulously scrutinized and submitted to a senior oncologist for expert adjudication.

Statistical analyses

The demographic and socioeconomic characteristics of cancer patients were described using frequencies and percentages. Consultation durations were demonstrated as mean and standard deviation. In the univariate analysis, the duration of consultation, the patient's age group (<60 years or ≥60 years), gender, ethnicity (Han or minority), marital status (married or single/widowed/divorced), location of registered residence (rural or urban), presence of caregivers during consultations (yes or no), the timing of their outpatient appointments (from 8:00 a.m. to noon or from 2:00 p.m. to 6:00 p.m.), and physician's gender were subjected to Student's t-test. The patient's highest education level (below senior school or high school and above), disease metastasis status (no metastasis or metastasis), and current receipt of anti-tumor treatment (yes or no) were assessed using the Mann-Whitney U test. The patient's medical insurance type (Urban Resident-based Basic Medical Insurance, Urban Employee Basic Medical Insurance, self-financed medical care, or New Rural Cooperative Medical Scheme/social insurance), number of chronic diseases (0, 1, 2, or ≥3), and years of experience in physician outpatient work (<10 years, 10-20 years, or >20 years) were examined using one-way analysis of variance. The type of malignant tumors (ICD-10 classification) and different consultation contents were evaluated using the Kruskal-Wallis H test. Multiple linear regression was used to determine factors affecting the duration of consultations. We examined the assumptions of the model and applied a logarithmic transformation to the outcome variable to facilitate linear regression analysis (Appendix 3, 4, 5). Variables demonstrating statistically significant differences (p<0.05) were retained for multiple linear regression models to ascertain their influence. Five variables were included in the analysis: (1) presence of caregivers during consultations (yes or no); (2) type of malignant tumors (primary malignancies located in the digestive organs (C15-C26); malignant tumors of the respiratory and intrathoracic organs (C30-C39); malignant tumors of the female genitals (C51-C58); malignant tumors of the lip, oral cavity, and pharynx (C00-C14); malignant tumors of the male genitals (C60-C63); malignant tumors of lymphoid, hematopoietic, and related tissues (C81-C96), or others); (3) presence of distant metastasis of malignant tumors (yes or no); (4) the contents of doctor-patient communication (routine outpatient follow-up evaluation, administering the formalities requisite for patient admission, prescribing anti-tumor drugs, symptom control, medical insurance reimbursement, and other); and (5) years of experience in physician outpatient work (<10, 10-20, or >20 years). All statistical analyses were performed using IBM SPSS Statistics for Windows, Version 26 (Released 2019; IBM Corp., Armonk, New York, United States), and two-sided p-values of <0.05 were considered statistically significant.

## Results

Demographic and clinical features

The average age of 620 cancer patients was 58.89 years. Among them, 354 were males, 614 were Han Chinese, 583 were married, 466 were educated below high school, 294 were rural residents, and 346 were accompanied by caregivers during consultations, and the appointment time was from 8:00 a.m. to 12:00 p.m. In addition, 309 patients had primary malignancies located in the digestive organs, and 258 patients were not currently receiving antitumor therapy (Table 1).

**Table 1:** Demographic and clinical characteristics of the 620 cancer patients

Characteristics	Number（%）
Age	
<60	329 (53.06)
≥60	291 (46.94)
Sex	
Male	354 (57.10)
Female	266 (42.90)
Ethnicity	
Han	614 (99.03)
Ethnic minority	6 (0.97)
Marital status	
Married	583 (94.03)
Single	10 (1.61)
Widowed	15 (2.42)
Divorced	12 (1.94)
Highest educational level	
Below senior school	466 (75.16)
High school and above	154 (24.84)
Location of registered residence	
Rural	294 (47.42)
Urban	326 (52.58)
Type of health insurance	
Urban Resident-based Basic Medical Insurance	471 (75.97)
Urban Employee Basic Medical Insurance	112 (18.06)
New Rural Cooperative Medical Scheme	4 (0.66)
Self-financed medical care	29 (4.68)
Social insurance	4 (0.66)
Presence of caregivers during consultations	
No	274 (44.19)
Yes	346 (55.81)
The timing of their outpatient appointments	
From 8:00 a.m. to 12:00 p.m.	456 (73.55)
From 2:00 p.m. to 6:00 p.m.	164 (26.45)
Type of malignant tumors	
Primary malignancies located in the digestive organs (C15–C26)	309 (49.84)
Malignant tumors of the respiratory and intrathoracic organs (C30–C39)	140 (22.58)
Malignant tumors of the female genitals (C51–C58)	67 (10.81)
Malignant tumors of the lip, oral cavity, and pharynx (C00–C14)	36 (5.81)
Malignant tumors of the male genitals (C60–C63)	16 (2.58)
Malignant tumors of lymphoid, hematopoietic, and related tissues (C81–C96)	16 (2.58)
Malignant tumors of the urinary tract (C64–C68)	8 (1.29)
Malignant tumors of the breast (C50)	8 (1.29)
Melanoma and other malignant tumors of the skin (C43–C44)	5 (0.81)
Malignant tumors of the eye, brain, and other parts of the central nervous system (C69–C72)	4 (0.65)
Malignant tumors of the thyroid and other endocrine glands (C73–C75)	3 (0.48)
Malignant tumors of the bones and articular cartilages (C40–C41)	3 (0.48)
Malignant neoplasms of independent (primary) multiple sites (C97)	3 (0.48)
Malignant tumors of mesothelial and soft tissues (C45–C49)	2 (0.32)
Chronic condition count	
0	387 (62.42)
1	161 (25.97)
2	45 (7.26)
≥3	27 (4.35)
Status of distant metastasis of malignant tumors	
The tumor has not spread to distant sites	339 (54.68)
The tumor has metastasized to distant sites	281 (45.32)
Type of ongoing treatment received	
Targeted therapy	168 (27.10)
Chemotherapy	161 (25.97)
Immunotherapy	51 (8.23)
Radiotherapy	34 (5.48)
Endocrine therapy	12 (1.94)
No treatment	258 (41.61)

The content of doctor-patient communication and consultation hours

**Figure 1:**
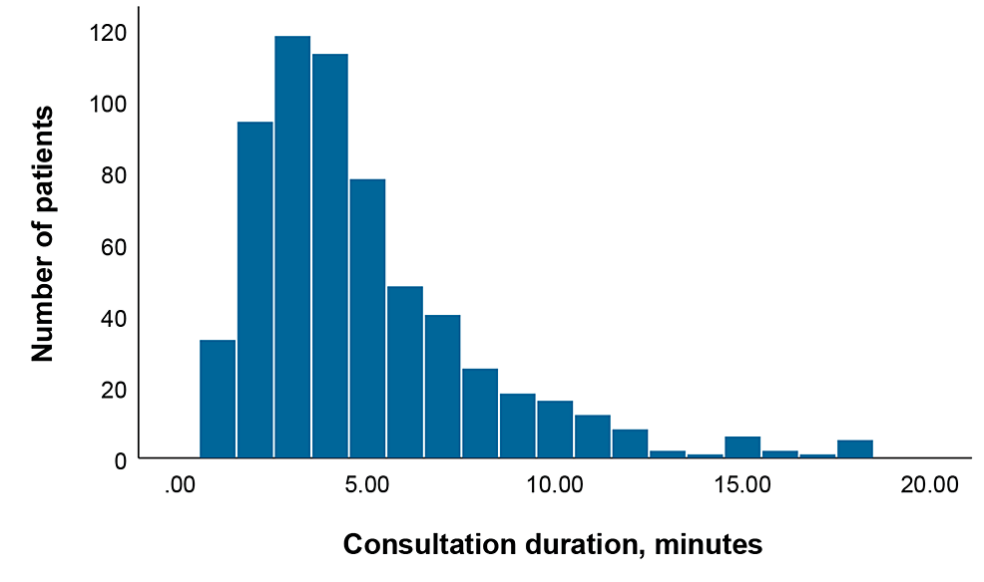
Histogram of consultation duration for 620 cancer patients

We divide the main content of doctor-patient communication during the outpatient visit into six categories. The duration of consultations for 620 patients is presented in histogram form (Figure 1). The mean consultation time for 620 cancer patients was 4.85 minutes (standard deviation of 3.09 minutes). Routine outpatient follow-up was the main communication content of 182 patients, with a mean duration of 3.81 minutes (standard deviation of 2.24 minutes). In 51 patients, the main content of communication was about admission procedures, and the mean duration of the visit was 3.00 minutes (standard deviation of 1.92 minutes). For 67 patients, the primary communication involved the prescribing of antineoplastic agents, with a mean consultation time of 3.70 minutes (standard deviation of 2.30 minutes). Symptom management discussions were the main content of communication, with a total of 107 patients and a mean of 5.91 minutes (standard deviation of 3.44 minutes). The main communication content of the 24 patients related to medical insurance reimbursement was 7.75 minutes (standard deviation of 4.63 minutes) (Table 2).

**Table 2:** The main content of doctor-patient communication and the duration of consultation in 620 cancer patients SD: standard deviation; CI: confidence interval

Main contents of doctor-patient communication	Number（%）	Consultation duration, mean (SD)	95% CI
Total	620 (100)	4.85 (3.09)	4.60 to 5.09
Routine outpatient follow-up evaluation	182 (29.35)	3.81 (2.24)	3.48 to 4.13
Administering the formalities requisite for patient admission	51 (8.23)	3.00 (1.92)	2.46 to 3.54
Prescribing anti-tumor drugs	67 (10.81)	3.70 (2.30)	3.14 to 4.26
Symptom control	107 (17.26)	5.91 (3.44)	5.25 to 6.57
Medical insurance reimbursement	24 (3.87)	7.75 (4.63)	5.80 to 9.70
Others	189 (30.48)	5.78 (3.10)	5.33 to 6.22

Influencing factors related to the duration of the consultation

Univariate analysis of the duration of counseling for cancer patients is provided in the supplementary material (Appendix 2). Multiple linear regression results showed that the duration of consultations with a caregiver was longer than that with no caregiver (b=0.12, 95% confidence interval (CI), 0.06 to 0.24). The duration of consultation was shorter in women with primary genital malignancies compared with the total duration of consultation in patients with primary gastrointestinal malignancies (b=-0.11, 95% CI, -0.37 to -0.07). Patients with distant metastases had a longer consultation time compared with those without distant metastases (b=0.12, 95% CI, 0.05-0.23). Compared with the consultation for routine outpatient follow-up assessment, the consultation time for symptom control and medical insurance reimbursement was also longer (symptom control: b=0.22, 95% CI, 0.22-0.50, medical insurance reimbursement: b=0.16, 95% CI, 0.25-0.70, others: b=0.26, 95% CI, 0.23-0.46). The duration of the consultation (including procedures required for admission) was shorter than that of the routine outpatient follow-up assessment (b=-0.12, 95% CI, -0.44 to -0.10) (Table 3).

**Table 3:** Multiple linear regression analysis showing factors associated with variation in oncology outpatient consultation durations Ref: reference; SE: standard error; CI: confidence interval Note: In the linear regression model, the R is 0.47, the R-squared is 0.22, the R-squared change is 0.20 (P＜0.001), and the F-value is 11.42(P＜0.001). *p<0.05, **p<0.01, ***p<0.001; C15-C26: primary malignancies located in the digestive organs; C30-C39: malignant tumors of the respiratory and intrathoracic organs; C51-C58: malignant tumors of the female genitals; C00-C14: malignant tumors of the lip, oral cavity, and pharynx; C60-C63: malignant tumors of the male genitals; C81-C96: malignant tumors of lymphoid, hematopoietic, and related tissues; others: malignant tumors of the urinary tract (C64-C68), malignant tumors of the breast (C50), melanoma and other malignant tumors of the skin (C43-C44), malignant tumors of the eye, brain, and other parts of the central nervous system (C69-C72), malignant tumors of the thyroid and other endocrine glands (C73-C75), malignant tumors of the bones and articular cartilages (C40-C41), malignant neoplasms of independent (primary) multiple sites (C97), malignant tumors of mesothelial and soft tissues (C45-C49).

Characteristic	B	HERSELF	b	t	95% CI
Presence of caregivers during consultations					
No (Ref.)					
Yes	0.15	0.05	0.12	3.31^**^	0.06 to 0.24
Type of malignant tumors					
C15–C26 (Ref.)					
C30–C39	0.00	0.06	0.00	0.001	–0.11 to 0.11
C51–C58	–0.22	0.08	–0.11	–2.94^**^	–0.37 to –0.07
C00–C14	–0.15	0.10	–0.06	–1.50	–0.34 to 0.05
C60–C63	0.09	1.15	0.02	0.64	–0.19 to 0.38
C81–C96	–0.11	0.14	–0.03	–0.78	–0.39 to 0.17
Others	0.10	0.10	0.04	1.00	–0.10 to 0.29
Status of distant metastasis of malignant tumors					
The tumor has not spread to distant sites. (Ref.)					
The tumor has metastasized to distant sites.	0.14	0.05	0.12	3.04^**^	0.05 to 0.23
Main contents of doctor-patient communication					
Routine outpatient follow-up evaluation (Ref.)					
Administering the formalities requisite for patient admission	–0.27	0.09	–0.12	–3.06^**^	–0.44 to –0.10
Prescribing anti-tumor drugs	–0.14	0.08	–0.07	–1.76	–0.30 to 0.02
Symptom control	0.36	0.07	0.22	5.18^***^	0.22 to 0.50
Medical insurance reimbursement	0.47	0.12	0.16	4.12^***^	0.25 to 0.70
Others	0.34	0.06	0.26	5.92^***^	0.23 to 0.46
Years of experience in physician outpatient work					
<10 years (Ref.)					
10–20 years	–0.10	0.05	–0.08	–1.82	–0.20 to 0.01
>20 years	0.05	0.07	0.03	0.77	–0.08 to 0.18

## Discussion

In this study, the mean duration of consultations for cancer patients was <5 min in the outpatient oncology clinic of a tertiary hospital in western China. The main contents of doctor-patient communication were divided into six categories: routine outpatient follow-up evaluation, administering the formalities requisite for patient admission, prescribing anti-tumor drugs, symptom control, medical insurance reimbursement, and others. Routine outpatient follow-up evaluations were common content in the outpatient doctor-patient communication. Less than one-fifth of doctor-patient communication involved symptom management. In addition, there was communication between doctors and patients about medical insurance reimbursement in the oncology outpatient clinic. Our results identified caregiver-accompanied consultations, malignant tumor types, distant metastases status, and different consultation contents as factors influencing the consultation duration.

Although there is a lack of research on the consultation duration and consultation content of oncology outpatient clinics in various countries, there are abundant research practices on general practice clinics in hospitals. The average consultation duration in the general practice clinic of the hospital varies worldwide, ranging from 48 seconds in Bangladesh to 22.5 minutes in Sweden. Approximately 18 countries, representing approximately 50% of the global population, spend <5 min with a primary healthcare practitioner, which is close to our results [14]. A study from community hospitals in two urban areas of Beijing had a median consultation duration of two minutes for general practice outpatient [15], and our results differed significantly from theirs. In terms of the outpatient volume, our study was similar to that of a Beijing community hospital, with each outpatient physician handling approximately 40 patients per half-day appointment. Discrepancies occurred in the study populations. The population selected for the study from the community hospital in Beijing was chronically ill, and because outpatient follow-up management of chronic diseases is more mature and accessible to the population, patient consultation durations are relatively short [16]. In contrast, the population selected for our study were cancer patients, and because of the complexity of cancer, outpatient physicians need sufficient time to determine the patient's disease status to prevent more serious complications.

A study delineated 10 healthcare service options for oncology outpatient care, comprising prevention, screening, and diagnosis; chemotherapy; radiotherapy; blood component therapy; patient and family education; discharge planning and referrals; nutritional support; group/individual counseling; physical therapy/rehabilitation; and outpatient surgery [17]. A study in Beijing classified the consultation contents of general practice clinics into six categories: specific prescription, presenting with symptoms, check-up, counseling, transfusion & injection, and sickness certificate [15]. The categories of symptom control and medical insurance procedure in our study were similar to the categories of presenting symptoms and sickness certificate in Beijing general practice clinics. Because antineoplastic drugs are usually required by mouth or injection, we combined the specific prescription with the transfusion & injection category and extended it to the prescribing anti-tumor drugs category. We extended the check-up category to the routine follow-up category according to the actual situation of the outpatient oncology clinic.

The presence of caregivers during patient consultations was associated with extended consultation durations. The emergence of caregivers inevitably increases expressive needs, thus inherently complicating the outpatient setting [18]. Findings from a clinic in Singapore showed that, on average, 72% of 281 cancer caregivers reported involvement in the decision-making process [19]. The caregivers in a patient's healthcare journey can facilitate the physicians' comprehension and identification of the patient's objectives, requirements, preferences, and principles [18]. The delivery of health care in outpatient oncology departments requires careful consideration of caregiver involvement in the care of cancer patients. This warrants further refinements of tailored and standardized approaches, such as shared decision-making tools [20], to better cater to the needs of outpatient oncology clinics.

In this study, female patients with genital malignancies were at a disadvantage in terms of outpatient consultation duration. Female genital problems prolong the duration of general practice outpatient consultations in Australia [21]. However, the female genital problems in this study did not include cancers of the female genital system. Inadequate knowledge of the disease, sex inequality, and discrimination may limit their ability to access and receive high-caliber cancer treatment [22]. Notably, cervical cancer, one of the prevalent primary malignant neoplasms of the female genital tract, is associated with sexually transmitted infections and has insidious symptoms. Women grappling with cervical cancer often exhibit reluctance to discuss their symptoms or seek treatment owing to feelings of self-reproach and disgrace [23]. In addition to drawing the attention of outpatient oncologists to female patients with genital malignancies, it is imperative to institute an accessible, inclusive healthcare framework that furnishes dignified, superior medical attention to the female cancer demographic.

In our study, consultation contents about routine outpatient follow-up evaluation, administering the formalities requisite for patient admission, and prescribing anti-tumor drugs were easy to identify because patients only wanted the oncologist to provide prescriptions and check in; the oncologist did not do anything else beyond that. The focus of conversations in all three categories is on prescribing and evaluating the results of various tests. Regression analysis showed that the outpatient consultation time of cancer patients who only came for admission procedures was shorter, because the admission process was simpler, and doctors may also prefer simplified communication strategies in the outpatient environment compared with the doctor-patient interaction after the patient was hospitalized [24].

Among the 620 outpatient consultations by cancer patients, 24 patients communicated with their oncologists about reimbursement procedures, which typically involve patients requesting medical claims, often handwritten, or typed by physicians. Such patterns present discrepancies compared to outpatient practices in other countries. The reimbursement procedure places serious time demands on clinicians because their time is devoted to completing paperwork as opposed to direct patient care [25]. A study from China found that 38.04% of the four-minute outpatient consultation time was spent on paperwork [26]. We observed that the reimbursement process, while bringing convenience to outpatients with cancer, also sacrifices the physicians' time that should be devoted to medical care. It is advisable to address these intricate issues before the arrival of patients, a responsibility that typically falls into the hands of front-office staff (or office support personnel). This approach saves the outpatient from wasting time in the office waiting for documents to arrive.

Symptom management was also one of the services that needed to be provided in oncology outpatient clinics. Among 620 cancer patients, 362 patients were currently receiving anti-tumor treatment, but only 107 patients had any communication with their doctor about symptom control. The study showed that most outpatient cancer patients who received chemotherapy reported severe symptoms [10]. This suggests that outpatients who do not communicate their symptoms with their physicians may have symptoms that are not recognized by outpatient physicians. In addition, information from oncology outpatient consultations is organized hierarchically for treatment decisions rather than primarily for symptom management. Only when clinically clear information is not available does the physician ask about symptoms [27]. Research indicates that owing to the substantial patient volume at oncology outpatient clinics in China, many cancer patients opt to seek treatment in the emergency department to address their symptoms when they are unable to secure an appointment at the oncology outpatient clinic [28]. How to obtain and manage the symptoms of cancer patients is an urgent clinical problem to be solved. Outpatient cancer patients have been shown to benefit from the assessment of symptoms by a patient-reported outcomes (PRO) tool prior to presentation [10]. In a region of Sweden, cancer patients reported and measured their health status and feelings by clicking on a screen before visiting their outpatient doctor [29]. Outpatient physicians can pre-plan the duration and content of their work (whether for emergency care or regular follow-ups) under the guidance of patient needs and priorities [30]. Therefore, in oncology outpatient clinics in China, patients using the PRO tool to report symptoms to doctors before their consultation may improve outpatient symptom management services.

Our study has some limitations. First, limitations of a single-center study are unavoidable, and the generalizability of our results to hospital outpatient clinics in different regions is limited. Second, our observational study was cross-sectional and thus unable to capture the duration of multiple clinic visits for individual patients, which was not the aim of this study but could represent the outpatient service burden. Third, a sufficient sample size of outpatient oncologists is required to assess the effect of physician-related factors on consultation duration. Owing to the study limitations, we were unable to identify a sufficiently large number of physicians during the study period. Fourth, we have narrowly focused on consultation duration without analyzing the quality of communication. Higher quality of doctor-patient communication may also have an impact on the length of consultations. Fifth, we did not investigate the satisfaction of outpatient cancer patients and oncologists with consultation duration and lacked the perspective of both doctors and patients to improve the efficiency of follow-up in oncology outpatient clinics. Last, we only made a simple classification of the communication content between doctors and patients in the oncology outpatient clinics of public hospitals and did not further distinguish the independent consultation time of each category.

In conclusion, the special needs of outpatient follow-up of cancer patients can be reflected in profiles by measuring the communication content that busy outpatient cancer patients communicate with their doctors during routine follow-up. We highlighted the need to reallocate staffing resources, especially for the most time-consuming issue, insurance reimbursement documentation. In addition, the outpatient departments of public hospitals in China do not pay enough attention to cancer treatment-related symptoms, and more efficient outpatient management strategies are needed.

## Conclusions

This study demonstrated the main content and duration of doctor-patient communication in the oncology outpatient clinics of public medical institutions in China. In outpatient cancer care, the conversation between oncologists and cancer patients tends to be monotonous and standardized, especially for cancer patients who are followed after hospital discharge. In addition to the medical consultations that outpatient physicians must perform, they are also responsible for spending additional time dealing with complex medical insurance reimbursement procedures. The apparent limited conversational interaction between physician and patient regarding symptom management calls into question whether this allocated consultation time is sufficient to thoroughly and effectively correct patients' symptoms. The health care administrations must devise reforms to balance the demand for oncology outpatient practitioners to provide effective outpatient care for recovering cancer patients. In addition, physicians in oncology outpatient clinics should pay attention to cancer patients who underwent admission procedures in the outpatient clinic and follow-up patients with gynecological malignancies. Future studies could systematically analyze and identify the multiple reasons for shorter outpatient consultation durations in patients with female genital malignancies.

## Figures and Tables

**Figure 2:**
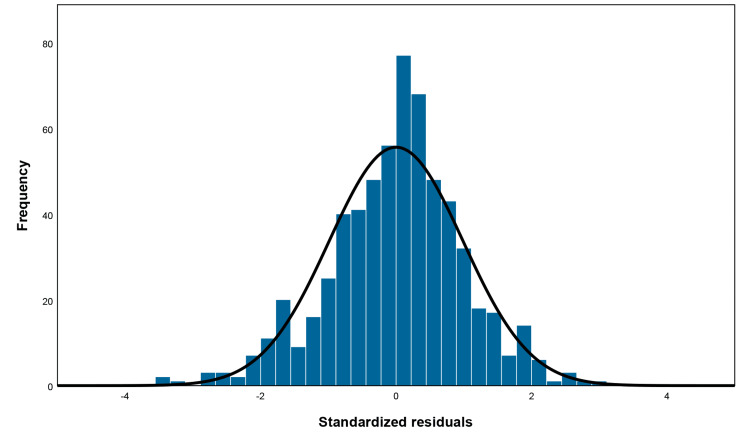
Histogram of the residuals

**Figure 3:**
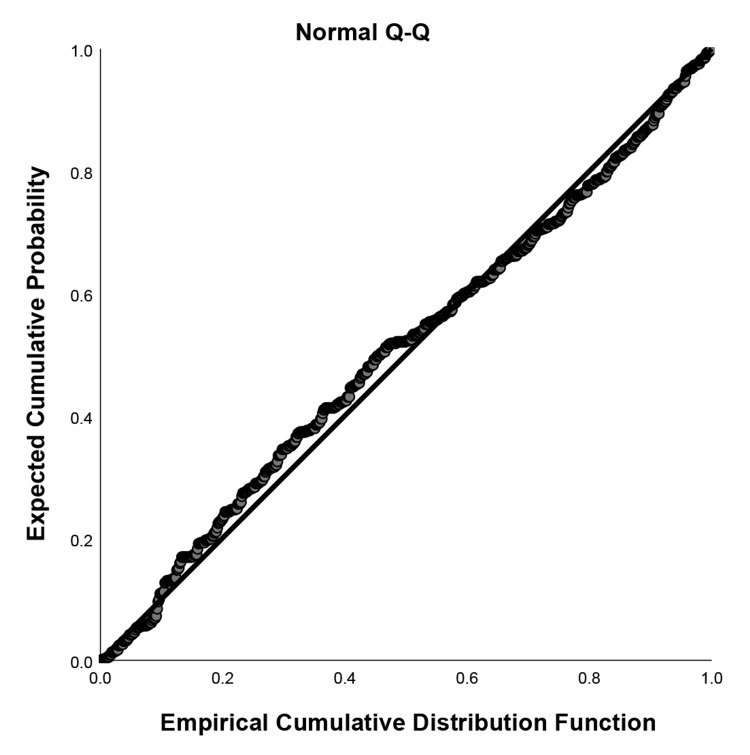
Residual Q-Q plot

**Figure 4:**
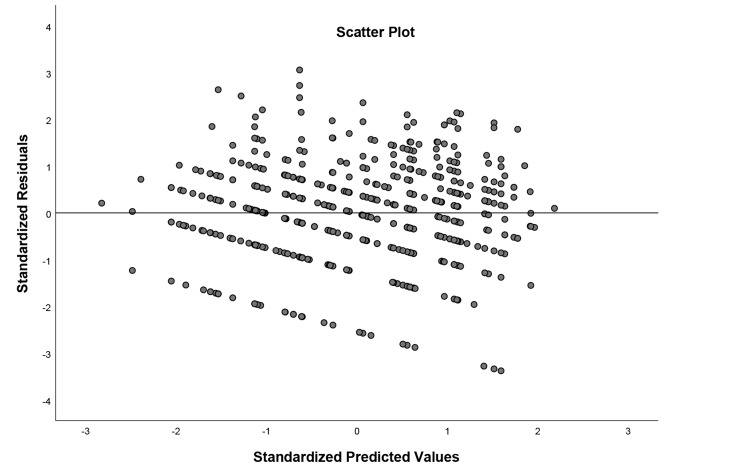
Plot of standardized residuals

## Appendices

Appendix 1

We added information about the eight oncology clinic physicians, as shown in Appendix 1.

**Table 4:** Detailed information on the eight clinicians

Characteristics	Categories	Frequency	Percentage
Sex			
	Male	6	75.00
	Female	2	25.00
Years of outpatient work			
	<10	2	25.00
	10–20	4	50.00
	>20	2	25.00
Outpatient work scheduling			
	From 8:00 a.m. to 12:00 p.m.	6	75.00
	From 2:00 p.m. to 6:00 p.m.	2	25.00

Appendix 2

The univariate analysis process and results affecting the consultation duration of outpatient cancer patients, as shown in Appendix 2.

**Table 5:** Univariate analysis of consultation duration in 620 cancer patients a: student’s t-test; b: Mann–Whitney U test; c: one-way analysis of variance; d: Kruskal-Wallis H test; N/A: not applicable

Variables	Mean (SD)	95% CI	Statistics	P
Age group				
<60 years	4.86 (3.25)	4.51 to 5.22	0.15^a^	0.877
≥60 years	4.82 (2.92)	4.49 to 5.16		
Sex				
Male	4.97 (3.32)	4.62 to 5.32	1.15^a^	0.251
Female	4.68 (2.77)	4.35 to 5.01		
Ethnicity				
Han	4.83 (3.07)	4.59 to 5.08	0.92^a^	0.359
Ethnic minority	6.00 (5.55)	N/A		
Marital status				
Married	4.86 (3.11)	4.61 to 5.11	0.47^a^	0.642
Single/widowed/divorced	4.63 (2.73)	3.75 to 5.50		
Highest educational level				
Below senior school	4.78 (2.96)	4.51 to 5.05	–0.15^b^	0.882
High school and above	5.04 (3.46)	4.49 to 5.59		
Location of registered residence				
Rural	4.70 (3.06)	4.35 to 5.05	1.10^a^	0.272
Urban	4.98 (3.13)	4.64 to 5.32		
Type of health insurance				
Urban Resident-based Basic Medical Insurance	4.83 (3.11)	4.55 to 5.11	0.65^c^	0.581
Urban Employee Basic Medical Insurance	5.04 (3.21)	4.42 to 5.63		
Self-financed medical care	4.79 (2.64)	3.79 to 5.80		
New Rural Cooperative Medical Scheme/social insurance	3.50 (2.07)	1.77 to 5.23		
Presence of caregivers during consultations				
No	4.40 (3.00)	4.04 to 4.76	–3.20^a^	0.001
Yes	5.20 (3.13)	4.87 to 5.53		
The timing of their outpatient appointments				
From 8:00 am to 12:00 p.m.	4.74 (3.01)	4.46 to 5.01	–1.45^a^	0.146
From 2:00 p.m. to 6:00 p.m.	5.15 (3.30)	4.64 to 5.66		
Type of malignant tumors				
Primary malignancies located in the digestive organs (C15–C26)	4.96 (3.22)	4.60 to 5.33	22.21^d^	0.001
Malignant tumors of the respiratory and intrathoracic organs (C30–C39)	5.03 (3.24)	4.49 to 5.57		
Malignant tumors of the female genitals (C51–C58)	4.04 (2.43)	3.45 to 4.64		
Malignant tumors of the lip, oral cavity, and pharynx (C00–C14)	3.86 (1.88)	3.22 to 4.50		
Malignant tumors of the male genitals (C60–C63)	6.75 (4.02)	4.61 to 8.89		
Malignant tumors of lymphoid, hematopoietic, and related tissues (C81–C96)	3.13 (1.15)	2.52 to 3.74		
Others	5.50 (2.83)	4.54 to 6.46		
Chronic condition count				
0	4.74 (3.01)	4.44 to 5.04	0.38^c^	0.769
1	4.99 (3.26)	4.48 to 5.50		
2	5.09 (3.25)	4.09 to 6.09		
≥3	5.03 (3.16)	3.79 to 6.29		
Status of distant metastasis of malignant tumors				
The tumor has not spread to distant sites	4.42 (2.86)	4.11 to 4.72	–3.79^b^	0.000
The tumor has metastasized to distant sites	5.36 (3.29)	4.98 to 5.75		
Current receipt of anti-tumor treatment				
No	4.58 (2.72)	4.24 to 4.91	–1.89^b^	0.060
Yes	5.05 (3.33)	4.69 to 5.38		
Main contents of doctor-patient communication				
Routine outpatient follow-up evaluation (Ref.)	3.81 (2.24)	3.48 to 4.13	78.57^d^	0.000
Administering the formalities requisite for patient admission	3.00 (1.92)	2.46 to 3.54		
Prescribing anti-tumor drugs	3.70 (2.30)	3.14 to 4.26		
Symptom control	5.91 (3.44)	5.25 to 6.57		
Medical insurance reimbursement	7.75 (4.63)	5.80 to 9.70		
Others	5.78 (3.10)	5.33 to 6.22		
Sex of physician				
Male	4.73 (3.01)	4.43 to 5.02	–1.26^a^	0.209
Female	5.05 (3.25)	4.63 to 5.48		
Years of experience in physician outpatient work				
<10 years	4.91 (2.93)	4.47 to 5.36	3.67^c^	0.026
10–20 years	4.59 (3.02)	4.26 to 4.91		
>20 years	5.48 (3.44)	4.85 to 6.11		

Appendix 3

Appendix 4

Appendix 5
